# 3D-Printed custom-made hemipelvic endoprosthetic reconstruction following periacetabular tumor resection: utilizing a novel classification system

**DOI:** 10.1186/s12891-024-07509-8

**Published:** 2024-05-16

**Authors:** Xin Hu, Minxun Lu, Yitian Wang, Yi Luo, Yong Zhou, Xiao Yang, Li Min, Chongqi Tu

**Affiliations:** 1 Department of Orthopedic Surgery and Orthopedic Research Institute, West China Hospital, Sichuan University, Chengdu, 610041 China; 2 Model Worker and Craftsman Talent Innovation Workshop of Sichuan Province, No. 37 Guoxue Road, Chengdu, Sichuan 610041 China; 3 National Engineering Research Center for Biomaterials, Sichuan University, Chengdu, Sichuan 610064 People’s Republic of China; 4 Provincial Engineering Research Center for Biomaterials Genome of Sichuan, Sichuan University, Chengdu, 610064 China

**Keywords:** 3D-printed, Prostheses, Hemipelvectomy, Classification

## Abstract

**Background:**

Customized 3D-printed pelvic implants with a porous structure have revolutionized periacetabular pelvic defect reconstruction after tumor resection, offering improved osteointegration, long-term stability, and anatomical fit. However, the lack of an established classification system hampers implementation and progress.

**Methods:**

We formulated a novel classification system based on pelvic defect morphology and 3D-printed hemipelvis endoprostheses. It integrates surgical approach, osteotomy guide plate and prosthesis design, postoperative rehabilitation plans, and perioperative processes.

**Results:**

Retrospectively analyzing 60 patients (31 males, 29 females), we classified them into Type A (15 patients: Aa = 6, Ab = 9), Type B (27 patients: Ba = 15, Bb = 12), Type C (17 patients). All underwent customized osteotomy guide plate-assisted tumor resection and 3D-printed hemipelvic endoprosthesis reconstruction. Follow-up duration was median 36.5 ± 15.0 months (range, 6 to 74 months). The mean operating time was 430.0 ± 106.7 min, intraoperative blood loss 2018.3 ± 1305.6 ml, transfusion volume 2510.0 ± 1778.1 ml. Complications occurred in 13 patients (21.7%), including poor wound healing (10.0%), deep prosthesis infection (6.7%), hip dislocation (3.3%), screw fracture (1.7%), and interface loosening (1.7%). VAS score improved from 5.5 ± 1.4 to 1.7 ± 1.3, MSTS-93 score from 14.8 ± 2.5 to 23.0 ± 5.6. Implant osseointegration success rate was 98.5% (128/130), with one Type Ba patient experiencing distal prosthesis loosening.

**Conclusion:**

The West China classification may supplement the Enneking and Dunham classification, enhancing interdisciplinary communication and surgical outcomes. However, further validation and wider adoption are required to confirm clinical effectiveness.

**Supplementary Information:**

The online version contains supplementary material available at 10.1186/s12891-024-07509-8.

## Background

Pelvic bone tumors represent a heterogeneous group of diseases with distinctive biological properties that can alter the appearance, structure, and inherent stability of the pelvic girdle; these tumors can range from benign to malignant and can be either primary or metastatic [1–3]. limb-salvage surgery, be it palliative or curative, plays a pivotal role in alleviating symptoms, achieving local control, and facilitating postoperative functional rehabilitation. However, the complex pelvic anatomy and tumor invasion make this process exceptionally difficult. Various reconstruction methods, both biological [4–7] and non-biological [8–11], have pros and cons, leading to choices being complicated due to the lack of consensus and ideal solutions. Among them, the 3D-printed custom-made hemipelvic endoprosthesis with a porous structure offers improved stability, cosmesis, faster recovery, and unique benefits like enhanced osseointegration and long-term stability [12–15]. This technique has gained significant attention in clinical practice due to improved functionality and reduced postoperative complications compared to traditional methods [16–25].

**Fig. 1:**
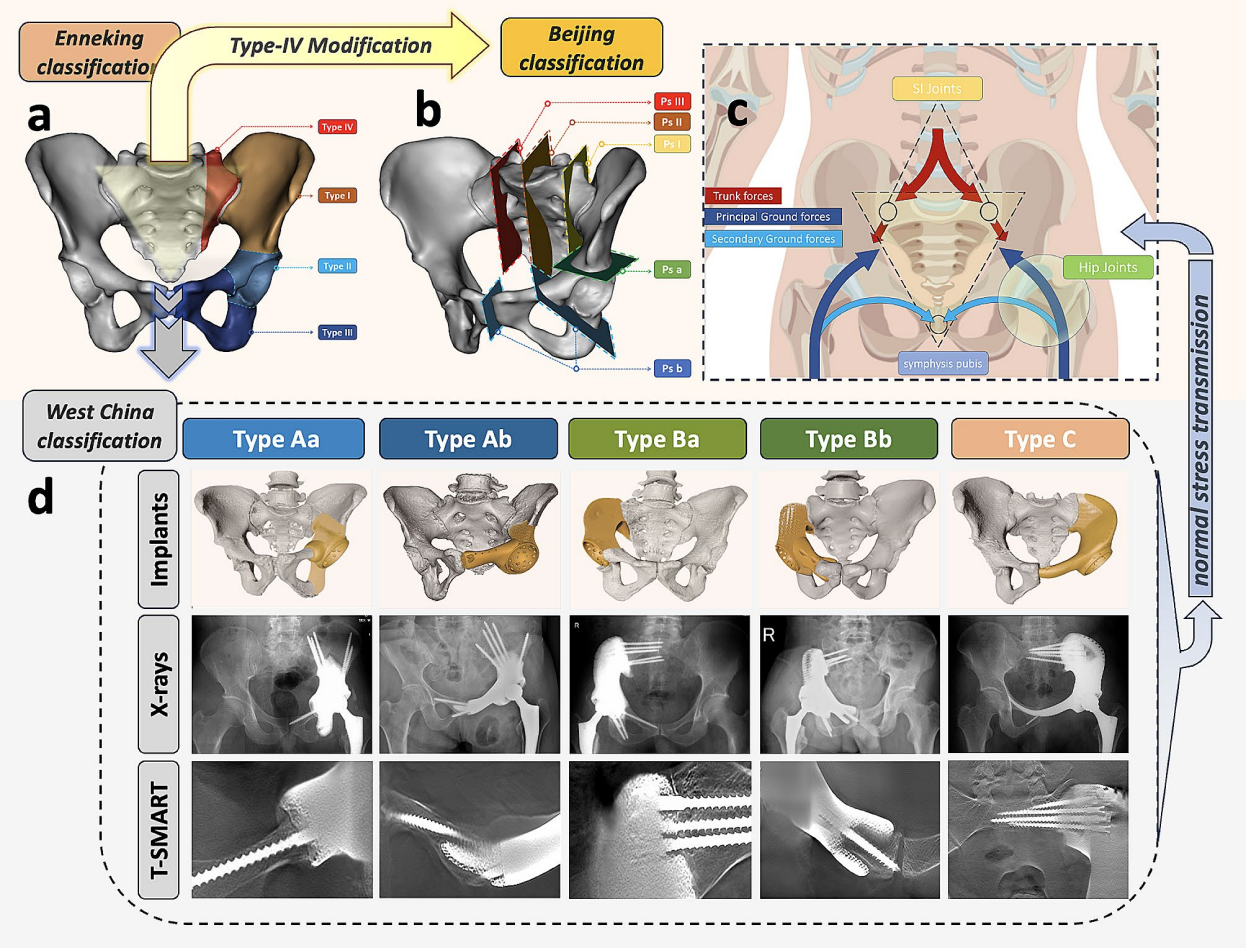
The underpinning of the West China Classification: **(a)** Enneking classification for pelvic resections: P1 (ilium), P2 (peri-acetabulum), P3 (pubis), and P4 (sacrum). **(b)** PKUPH classification for pelvic-sacral resections: P-s I (ipsilateral sacroiliac joint), P-s II (ipsilateral sacral foramina), P-s III (involvement lateral to contralateral sacral foramina), P-s a (absence of acetabular involvement), and P-s b (presence of acetabular involvement). **(c)** Physiological stress transmission in the pelvic ring: Arrows indicate body weight force direction within the pelvic ring, trunk, and femurs. Key anatomical structures for stress distribution: (1) Sacrum - keystone of the pelvic ring, wedged between ilia and secured by sacroiliac joints. (2) Hip joints bear downward stress. (3) Pubic symphysis connects pelvis into a circular structure. Reprinted with permission from Hu et al. [16] ©2023 British Journal of Surgery **(d)** West China Classification: 3D-printed pelvic prostheses were employed for Type A (hip joint loss), Type B (hip and sacroiliac joint loss), and Type C (hip, sacroiliac, and pubic symphysis joint loss) reconstructions after resecting typical pelvic tumors. Subtype a featured an intact obturator ring, while subtype b exhibited an incomplete obturator ring. T-smart imaging demonstrated favorable prosthetic-bone integration post-reconstruction

Surgical classification is essential for improving patient care and treatment planning. The Enneking and Dunham’s pioneering classification [26] (1978) for hemipelvic limb salvage surgery focused on osteotomy site but may need updates to accommodate modern reconstruction strategies (Fig. 1a). The Mayo classification [27] (2020) introduced a combined sacrectomy and hemipelvectomy approach, and a more recent sacropelvic classification [28] (2021) considered the extent of sacral resection (Fig. 1b). Nevertheless, these existing classifications lack sufficient consideration of vital anatomical structures within the pelvis, such as the hip joint, sacroiliac joint, pubic symphysis, and intact obturator ring (Fig. 1c), which significantly influence surgical complexity and outcomes. Recently, Kapoor et al. [29] emphasized the necessity of reassessing and refining the pelvic resection techniques outlined by Enneking. In their retrospective study of 82 pelvic tumor resections, preserving the acetabulum was correlated with significantly improved functional outcomes compared to complete acetabular resection. Furthermore, different types of resections led to varying functional outcomes, with transacetabular resections consistently exhibiting superior function while maintaining oncological efficacy. These findings underscore the necessity of reevaluating and refining pelvic resection techniques to optimize both functional recovery and oncological outcomes.

We sought to formulate a novel classification system of pelvic reconstruction using 3D-printed custom-made hemipelvic endoprosthesis, based on morphological characteristics of pelvic defects (Fig. 1d). Our study aims to propose this classification, describe surgical approaches and techniques for each subtype, and evaluate patient outcomes based on this novel classification system.

## Methods

### Patients

From April 2017 to January 2023, 111 patients with primary malignant pelvic girdle tumors were admitted, and the study included 60 of these patients. Inclusion criteria: (1) Pathological diagnosis of primary pelvic malignant tumor (2), No contraindication to en bloc resection (3), Reconstruction using 3D-printed custom-made integrated endoprostheses, and (4) Complete follow-up data. Exclusion criteria: (1) Unwillingness or inability to take risks of potential prosthetic complications (2), Presence of other serious diseases incompatible with anesthesia and surgery (3), Active infection around the prosthesis implantation site (4), Metal implant allergy (5), Lower limb deformities (affect subsequent MSTS lower limb functional assessment) (6), Severe osteoporosis (affects stability of prosthetic fixation), and (7) Incomplete follow-up data. Preoperatively, all patients underwent pathological examination for diagnosis and Enneking staging for tumor classification. Routine physical exams, biochemical analyses, and comprehensive imaging (X-ray, 3D-CT, MRI, SPECT) were conducted, including a thin-layer chest CT for possible lung metastases. Pre-surgery, VAS scores and MSTS-93 scale were evaluated and recorded.

The study adhered to the 1964 Helsinki Declaration and was approved by the Ethics Committee of West China Hospital. Written informed consent was obtained from adult participants or parents of minors (below 16 years of age).

### Novel classification system

This classification system uniquely combines pelvic defect morphology with 3D-printed custom-made hemipelvis endoprosthesis. It takes into account tumor resection scope and the loss of anatomical structures (hip joint, sacroiliac joint, pubic symphysis, and intact obturator ring). Extending the Enneking classification system, it categorizes cases into three types: Type A (hip joint loss), Type B (hip joint + sacroiliac joint loss), and Type C (hip joint + sacroiliac joint + pubic symphysis joint loss). Letters are used to distinguish it from the Enneking classification.

Furthermore, due to the influence of the obturator ring’s preservation on surgical approach and implantation difficulty, Type B was further subdivided into two categories: Type Ba (involving an intact ring) and Type Bb (involving an incomplete ring). In Type A, complete preservation of the obturator ring is infrequent. Consequently, it is subdivided based on the extent of anterior pelvic ring resection into Type Aa (intact pubic symphysis) and Type Ab (loss of pubic symphysis). The summarized tumor resection scope and key design considerations for each prosthesis type are presented in Table 1.

**Table 1:** Characteristics of Bone Resection, Implant Design, and Fixation Requirements for Different Types of Prostheses

Prosthesis Type	Anatomical structures lost	Integrity of the obturator	Integrity of the pubic symphysis	Screw quantity for stable prosthesis-bone fixation		Prosthesis design requirements	Surgical Approach
				Proximal interface	Distal interface		
Type Aa	• Hip joint	Intact	Intact	≧ 4	≧ 2	• Restore hip joint function	MGMII approach
Type Ab	• Hip joint	Incomplete	Intact/Incomplete	≧ 4	≧ 2		
Type Ba	• Hip joint • Sacroiliac joint	Intact	Intact	≧ 4	≧ 2	• Restore hip joint function • Effective sacroiliac joint fixation	Combined posterior iliac and Smith-Petersen approaches, optionally incorporating the ilioinguinal approach
Type Bb	• Hip joint • Sacroiliac joint	Incomplete	Intact	≧ 4	≧ 2		
Type C	• Hip joint • Sacroiliac joint • Pubic symphysis	Incomplete	Incomplete	≧ 4	Stem fixation	• Restore hip joint function • Effective sacroiliac joint fixation • Effective pubic symphysis fixation	MGMII approach

MGMII, combined and modified Gibson and ilioinguinal approach [31]

### Custom implant and osteotomy guide and surgical approach selection

**Fig. 2:**
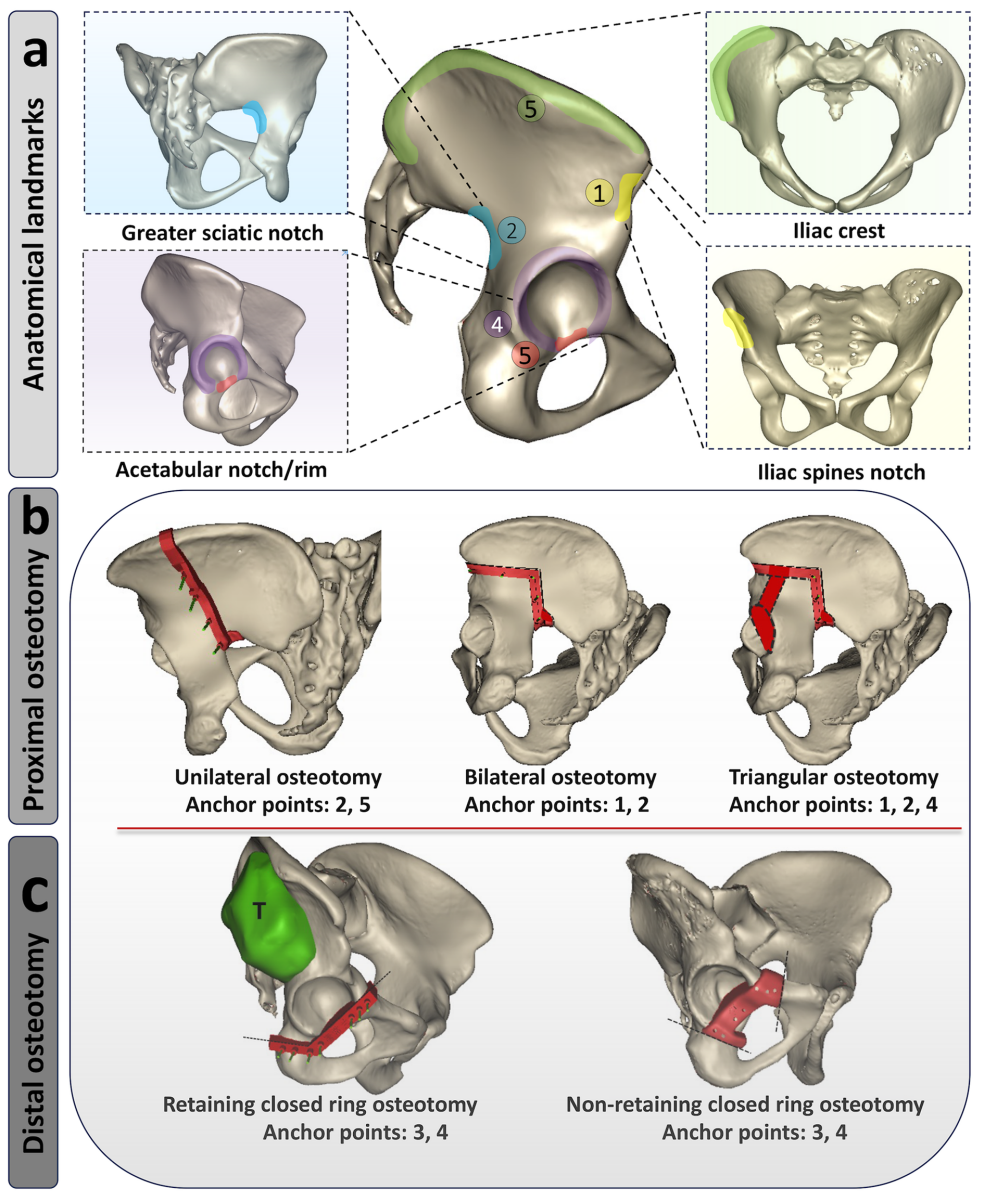
Customized osteotomy guide design for periacetabular tumor: **(a)** Summary of ideal anatomical landmarks for the pelvic upper osteotomy guide anchorage; **(b)** Summary of the proximal pelvic osteotomy approach; **(c)** Summary of the distal pelvic osteotomy approach

All prostheses were custom designed by our clinical team based on each patient’s last available imaging data before surgery. Firstly, based on our previous reports, the design and fabrication of the customized prosthesis were completed [30]. Secondly, the osteotomy range is carefully determined based on tumor margins, ensuring precise alignment of the guide with anchor points on the pelvis (Table 2). This classification includes proximal (e.g., sacroiliac joint region) and distal (acetabulum and pubic symphysis area) osteotomies, with corresponding guide designs (Fig. 2a-c). Thirdly, custom-designed endoprostheses addressed bony defects for each type, featuring porous and solid titanium (Ti6A14V) components. Acetabulum resection aimed for accurate hip joint replacement with proper rotation center, anteversion, and abduction angles using semi-porous acetabulum structure, proximal femoral head, and a constrained acetabular liner (15° anteversion, 45° inclination). Type B and C designs emphasized secure fixation with the sacral surface. Type B had supplementary screw holes aligned with the sacroiliac axis, while Type C utilized a pubic stem for stability at the pubic symphysis. Prosthesis design and fabrication took one to two weeks.

**Table 2:** Selection of Anchoring Points and Design Details of Bone Resection Guides on the Pelvis

Anchoring points	Anatomical landmarks	Anchoring requirements	Relevant surrounding blood vessels and nerves	Surgical site exposure requirements
1	Greater sciatic notch	Secure irregular notch, ≥ 15 mm contact width	Sciatic nerve	/
2	Acetabular notch	Fill irregular acetabular notch shape	/	Femoral head dislocation
3	Acetabulum rim	Hook acetabular contour/partially fill acetabular notch	Anterior acetabular rim: Femoral artery and vein, and femoral nerve	Femoral head dislocation/debridement of cartilage structures
4	Iliac crest	Hook the iliac crest edge	/	/
5	Anatomical notches at the anterior superior and inferior iliac spines	Hook the iliac crest edge	Femoral lateral cutaneous nerve (LFCN)	/

Of particular note is the considerable variation in the surgical field exposed during operations for different types of prosthetic reconstructions, owing to differences in the location and extent of tumor resection. Additionally, the design of the prosthesis must be aligned with the chosen surgical approach; for example, the direction of screw implantation must fall within the visible range of the established surgical approach to ensure seamless insertion. As a result, different prosthetic types correspond to distinct surgical approaches [31]. We summarize the selection of surgical approaches corresponding to various prosthetic reconstructions in Table 1.

### Postoperative management

#### Rehabilitation programs

After surgery, lower limbs were immobilized in specific positions (neutral rotation, 15° to 25° hip abduction, 15° hip flexion, and 15° knee flexion). Within three days, hip muscle strength was assessed using stability and extension tests previously reported for personalized rehab plan [30]. Patients passing both tests qualified for early rehab. At 3 days, gentle passive hip flexion and abduction exercises were given, and after 1 week, moderate weight-bearing was allowed with aids. Active hip exercises and single-leg stance were introduced at 2 weeks, and ambulation with aid was managed at 3 weeks. By the second month, some used canes for standing and walking, which were later removed, and squatting was allowed under supervision. However, patients failing one or both tests had poor hip stability or weak muscles, leading to an extension of bed rest and limb immobilization to 2–3 weeks. During this time, patients trained daily with the tests. Later, they were allowed to perform standing hip flexion without weight on the affected side. Partial weight-bearing was permitted at 5 weeks, gradually increasing. Hip exercises were encouraged, and walking with aids was initiated at 6–8 weeks post-op.

#### Follow-up routine

Clinical and radiological evaluations were followed up systematically at 1, 2, and 3 months, every 3 months for the first 2 years, and then every 6 months. Independent assessment by an unbiased surgeon. Specific indicators include:

- Surgical outcomes: Operation duration and blood loss.
- Pain and function: The VAS scores and the MSTS-93 scale were used to evaluate the pain relief and the lower-limb function at each follow-up.
- Complications: including infection, local recurrence, dislocation, aseptic loosening, endoprosthetic breakage, and delayed wound healing.
- Radiological outcome: Osteointegration was assessed using Tomosynthesis Shimadzu Metal Artefact Reduction Technology (T-SMART) [20, 25, 30–33]. The implant-host bone interfaces in all patients were analyzed.
- Oncological outcome: Evaluation of local recurrence, distant metastasis, and patient survival status.

### Statistical analysis

Independent-samples Student’s t-test for normally distributed data (operating time, intraoperative blood loss, VAS score, MSTS93 functional score). Mann-Whitney U test for non-normally distributed data. SPSS 21.0 was used for analysis (IBM Corp., Armonk, NY), and Prism software (GraphPad, La Jolla, CA) for graphical presentation. *p* < 0.05 (two-tailed test) considered statistically significant.

## Results

### Demographics

Examined 60 out of 111 consecutive patients (31 males, 29 females) with our novel surgical planning classification system. The demographic, classification data, and tumor data are summarized in Table 3. The remaining 51 patients were excluded for the following reasons: 4 lost to follow-up, 5 underwent hemipelvic amputation, and 6 chose modular hemipelvic prosthesis reconstruction due to time or financial constraints. Moreover, 36 patients who underwent Type I/I + IV/III resections with 3D-printed hemipelvic endoprosthetic reconstruction were excluded.

**Table 3:** Characteristics of patients undergoing hemipelvic replacement surgery

Characteristics	All patients (*N* = 60)
**Demographic**	
Sex*	
Male	31 (51.7%)
Female	29 (18.3%)
Age † (yr)	44.4 ± 15.9
BMI † (kg/m^2^)	23.7 ± 3.7
Follow-up time † (mo)	36.5 ± 15.0
Tumor histology*	
Chondrosarcoma	26 (43.3%)
Osteosarcoma	9 (15.0%)
Ewing sarcoma	8 (13.3%)
Renal clear cell carcinoma with pelvic metastases	2 (3.3%)
Lung adenocarcinoma with pelvic metastases	2 (3.3%)
Solitary plasmacytoma	2 (3.3%)
Synovial sarcoma	2 (3.3%)
Spindle cell carcinomas	1 (1.7%)
Myofibroblastic sarcoma	1 (1.7%)
Solitary fibrous tumor	1 (1.7%)
Colorectal adenocarcinoma with pelvic metastases	1 (1.7%)
Invasive chondroblastoma	1 (1.7%)
Metastatic squamous cell carcinoma	1 (1.7%)
Cervical cancer with pelvic metastases	1 (1.7%)
Malignant rhabdoid tumor	1 (1.7%)
Langerhans cell histiocytosis	
**Tumor volume (**Length x Width x Height, cm**)**	
Tumor length † (cm)	9.7 ± 3.6
Tumor width † (cm)	8.1 ± 3.3
Tumor height † (cm)	6.1 ± 3.1
Preoperative staging*	
IIB	51(85.0%)
III	9(15.0%)
**Neoadjuvant chemotherapy**	
No. of patients*	27(45.0%)
Reconstruction Classification*	
Type A(a + b)	15(25.0%)
Type Aa	6(10.0%)
Type Ab	9(15.0%)
Type B (a + b)	27(45.0%)
Type Ba	15(25.0%)
Type Bb	12(20%)
Type C	17(28.3%)
**Oncology outcomes (Status at time of latest follow-up)** *	
Local recurrence	4(6.7%)
No evidence of disease	52(86.7%)
Alive with disease	4(6.7%)
Died of disease	4(6.7%)

*The values are given as the number of patients, with the percentage in parentheses

†The values are given as the mean and the standard deviation

### Surgical outcomes and complications

**Fig. 3:**
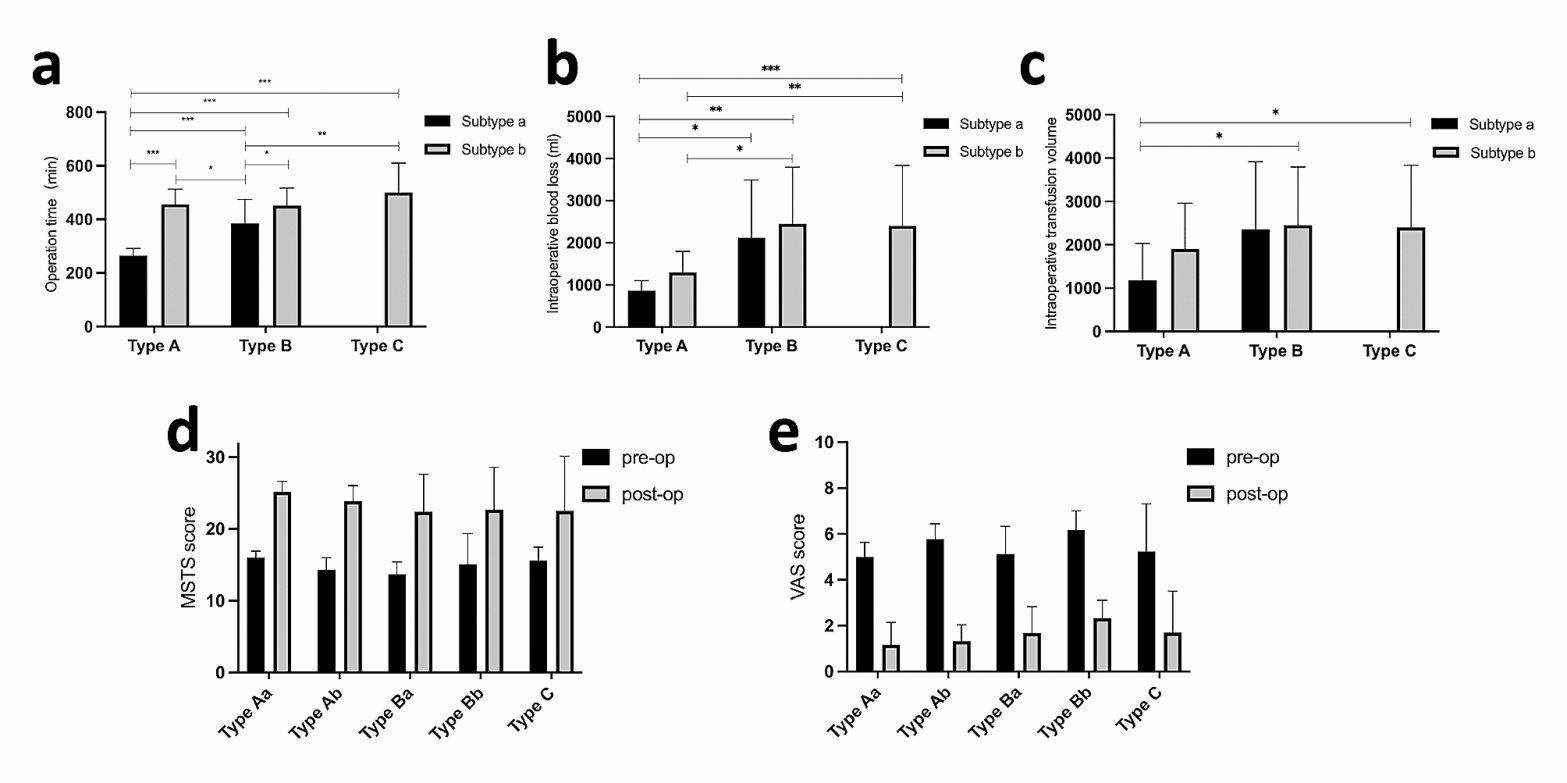
Surgical Outcomes and Functional Assessments: As the resection scope increases, there is a trend of prolonged surgical time **(a)**, intraoperative blood loss **(b)**, and transfusion volume **(c)**. Patients demonstrate significantly improved functional prognosis at the last follow-up after surgery, as evidenced by a significant increase in **(d)** MSTS scores and effective alleviation of **(e)** pain

Overall, the mean operating time for all patients from incision to wound closure was 430.0 ± 106.7 min (range, 236.0–680.0 min). The mean intraoperative blood loss and transfusion volume were 2018.3 ± 1305.6 ml (range: 500.0-6000.0 ml) and 2510.0 ± 1778.1 ml (range: 350.0-9700 ml), respectively. Type A group exhibited significantly lower intraoperative blood loss (1126.7 ± 457.4 ml), transfusion volume (1616.7 ± 1012.4 ml), and shorter surgery time (380.1 ± 106.8 min) compared to Type B (intraoperative blood loss: 2264.3 ± 1341.1 ml, transfusion volume: 2596.4 ± 1636.8 ml, surgery time: 414.3 ± 85.0 min) and Type C groups (intraoperative blood loss: 2400.0 ± 1435.7 ml, transfusion volume: 3155.9 ± 2244.2 ml, surgery time: 500.0 ± 108.8 min) (*P* < 0.05), indicating a trend of increasing blood loss and surgery time with larger resection scope. Furthermore, in both Type A and Type B groups, it was observed that the blood loss volume and surgical time in subgroup a were shorter than those in subgroup b (*P* < 0.05), indicating increased reconstruction difficulty when leaving an incomplete obturator ring after tumor resection. Additionally, Type C group had significantly higher blood loss and surgery time than Type A (*P* < 0.05), but no significant difference from Type B (*P* > 0.05) (Fig. 3a-c).

Intraoperative complications were absent, but postoperative complications occurred in 13 patients (21.7%), with no significant difference in the overall complication rate observed among the subtypes. The most prevalent complication observed was poor wound healing in six patients (10.0%). Among them, two patients received intensive wound dressing postoperatively for approximately two weeks, leading to wound closure upon discharge. Additionally, in four cases, intensive wound dressing was initiated postoperatively, followed by a subsequent procedure involving debridement under general anesthesia. During the procedure, necrotic and poorly healing tissues were excised, and the wound was repeatedly irrigated with 10% povidone-iodine solution and 3 L of isotonic sodium chloride solution via pulsatile lavage. Subsequently, vacuum-assisted closure (VAC) therapy was employed following debridement. Upon gradual improvement of the wound, the VAC drainage device was removed, and wound closure was achieved through meticulous wound care before discharge. In four instances (6.7%), deep prosthesis infection endured despite the diligent application of sustained Debridement, Antibiotics, and Implant Retention (DAIR) procedures. Despite conscientious efforts, the infection proved refractory, thereby necessitating the eventual removal of the implant as a last resort to achieve infection control.

Two cases had postoperative hip dislocation (3.3%), managed through closed reduction under anesthesia and stabilization with a T-shaped pillow and anti-rotation shoes, preventing further dislocation (Supplementary Fig. 1a). At the latest follow-up, no instances of dislocation were reported, with X-ray examinations revealing satisfactory positioning of the prosthesis. In addition, one Type Ba endoprosthesis had an upper sacroiliac joint screw fracture (1.7%) post-surgery, remaining asymptomatic without compromising prosthesis or limb function (Supplementary Fig. 1b). One Type Ba prosthesis exhibited distal-bone interface loosening (1.7%) and fractures of pubic and ischial screws after a 2-year follow-up. Subsequently, the patient underwent revision surgery, leading to a notable improvement in lower limb function postoperatively.

### Pain and lower extremity function

Across all patients, the average VAS score improved significantly from 5.5 ± 1.4 points (2 to 8) preoperatively to 1.7 ± 1.3 points (0 to 6) at the latest follow-up. MSTS-93 score also improved from 14.8 ± 2.5 points (11 to 24) preoperatively to 23.0 ± 5.6 points (4 to 29) at the most recent follow-up (Fig. 3D). For the Type A group, the average VAS score before and after surgery were 5.5 ± 0.7 and 1.3 ± 0.8, respectively; the MSTS-93 scores before and after surgery were 15.0 ± 1.6 and 24.4 ± 2.0, respectively. For the Type B group, the average VAS score before and after surgery were 5.6 ± 1.2 and 2.0 ± 1.0, respectively; the MSTS-93 scores before and after surgery were 14.3 ± 3.1 and 22.5 ± 5.4, respectively. For the Type C group, the average VAS score before and after surgery were 5.2 ± 2.1 and 1.7 ± 1.8, respectively; the MSTS-93 scores before and after surgery were 15.6 ± 1.9 and 22.5 ± 7.6, respectively.

The comparison among the three groups reveals that, at the final follow-up, Type A group had significantly lower VAS scores than Group B (*P* < 0.05), while MSTS scores were comparable; Type A group showed no significant differences in VAS or MSTS scores compared to Type C group (*P* < 0.05). There were no significant differences between Type B group and Type C Group in VAS or MSTS scores (*P* < 0.05) (Fig. 3d-e).

### Radiographic outcomes

**Fig. 4:**
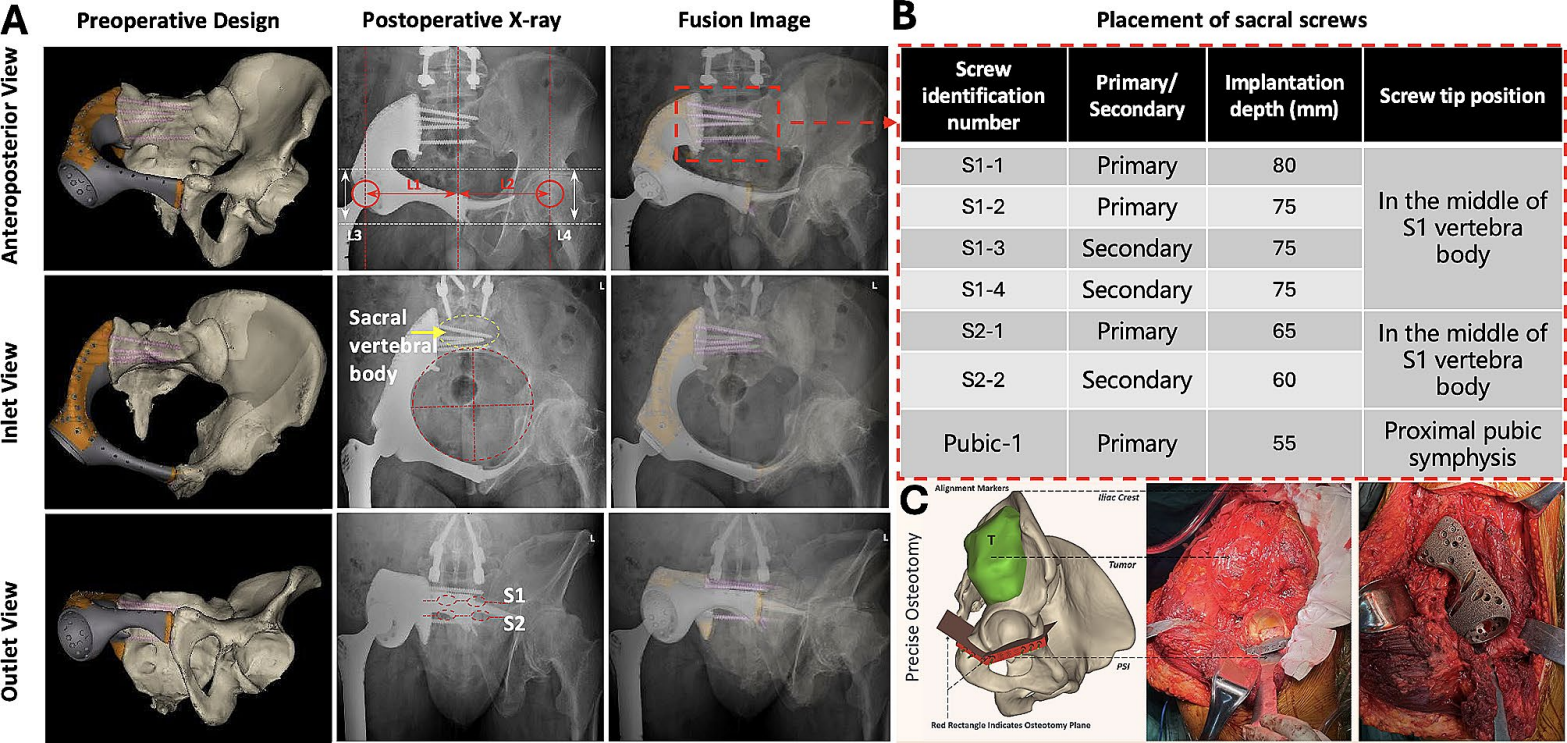
Illustration of the Type Ba reconstruction process: **(a)** Postoperative multi-directional pelvic X-ray assessment of the prosthesis implantation, demonstrating precise execution of osteotomy, prosthesis placement, and multi-level screw fixation. **(b)** Customized prosthetic screws are implanted with predetermined direction and depth. A total of 7 screw implantation channels are designated for the prosthetic, with 6 in the sacral region (4 in S1 vertebra, 2 in S2 vertebra) and 1 in the sacroiliac joint area. During surgery, priority is given to implanting the primary screw in the most optimal position, while secondary screws serve as alternative implantation plans. **(c)** Preoperative software-guided surgical simulation to define tumor resection margins, design osteotomy guides, and achieve accurate osteotomy and prosthesis implantation during surgery. Reprinted with permission from Hu et al. [16] ©2023 British Journal of Surgery

All patients underwent precise osteotomy, accurate prosthesis implantation, and planned screw fixation (position, quantity, and direction) according to the preoperative simulated surgery. In addition, except for one Type Ba patient experiencing distal prosthesis loosening, all other patients exhibited successful osseointegration of their implants during the final follow-up examination (T-SMART). Postoperative X-ray examinations revealed no evidence of bone absorption or osteolysis at the prosthesis-bone interface. The success rate of bone integration was calculated as 98.5% (128/130). A representative case is shown in Fig. 4a-c.

### Oncology outcomes

The mean follow-up duration was 36.5 ± 15.0 months. At the latest follow-up, 52 patients (86.7%) were found to have survived without any signs of disease recurrence. Four patients (6.7%) had succumbed to lung metastatic disease, indicating an average postoperative survival time of 7.25 ± 1.5 months (range: 6 to 9 months). Additionally, four patients (6.7%) were alive with local recurrence postoperatively, and their management involved either amputation (*n* = 3) or targeted therapy (*n* = 1).

## Discussion

A comprehensive classification system for pelvic resection in limb-salvage surgery serves as the foundation for reconstructing pelvic defects using 3D-printed hemipelvic endoprostheses. However, our classification system excludes Type I/I + IV and pure Type III resections due to ongoing controversies surrounding the necessity of surgical reconstruction [34–36]. Pure Type III resections, as they do not affect the primary weight-bearing axis of the pelvic ring, generally avoid pubic defect reconstruction to minimize complications [37, 38]. However, in the case of Iliac wing (Type I) and iliosacral (Type I + IV) pelvic resections, non-reconstruction can lead to the residual ilium collapsing onto the sacrum, forming an iliosacral pseudarthrosis that significantly compromises functionality [35]. Conversely, in cases involving tumor resection in the acetabulum region, most scholars agree that surgical reconstruction is necessary to restore stress transmission and hip joint function for limb preservation and better functional recovery. Hence, this new classification system is established based on the unique anatomical site of the acetabulum.

The classification system is based on the loss of periacetabular anatomical structures (obturator ring, pubic symphysis, and sacroiliac joint) after pelvic tumor resection. Overall, increasing resection extent (Type A to Type B to Type C) leads to more lost structures, resulting in higher intraoperative blood loss, transfusion volume, and surgical time, indicating increased surgical difficulty. Subtype a shows shorter surgical time and lower blood loss compared to subtype b. For example, type Bb exhibited a significant increase in surgical time and intraoperative blood loss compared to Type Ba subgroup. This difference can be attributed to the larger excision area required for the incomplete obturator ring in Type Bb cases, leading to extended surgical incisions and a higher risk of bleeding [31]. Moreover, the complex anatomy of the obturator ring deepens the bilateral osteotomy surfaces, posing challenges in tissue exposure and complicating the placement of osteotomy guide plates. Moreover, precise prosthesis fitting is demanded for both bilateral osteotomy surfaces, intensifying the complexity of prosthetic implantation. Additionally, the Type C group showed a broader resection extent compared to the Type B group. Surprisingly, no significant differences were observed in intraoperative blood loss and surgical duration between the two groups. This outcome may result from the simpler osteotomy procedure in the pubic symphysis region for Type C cases, facilitating a wider surgical exposure range and more efficient implementation of osteotomy guide plates and prosthesis placement. Furthermore, the limited number of screws required for fixation in the pubic symphysis region for Type C cases may have contributed to a shorter overall surgical time. Those results demonstrated that the refined subtypes offer a more comprehensive understanding of the anatomical variations and surgical complexities associated with tumor resections and reconstructive procedures.

**Fig. 5:**
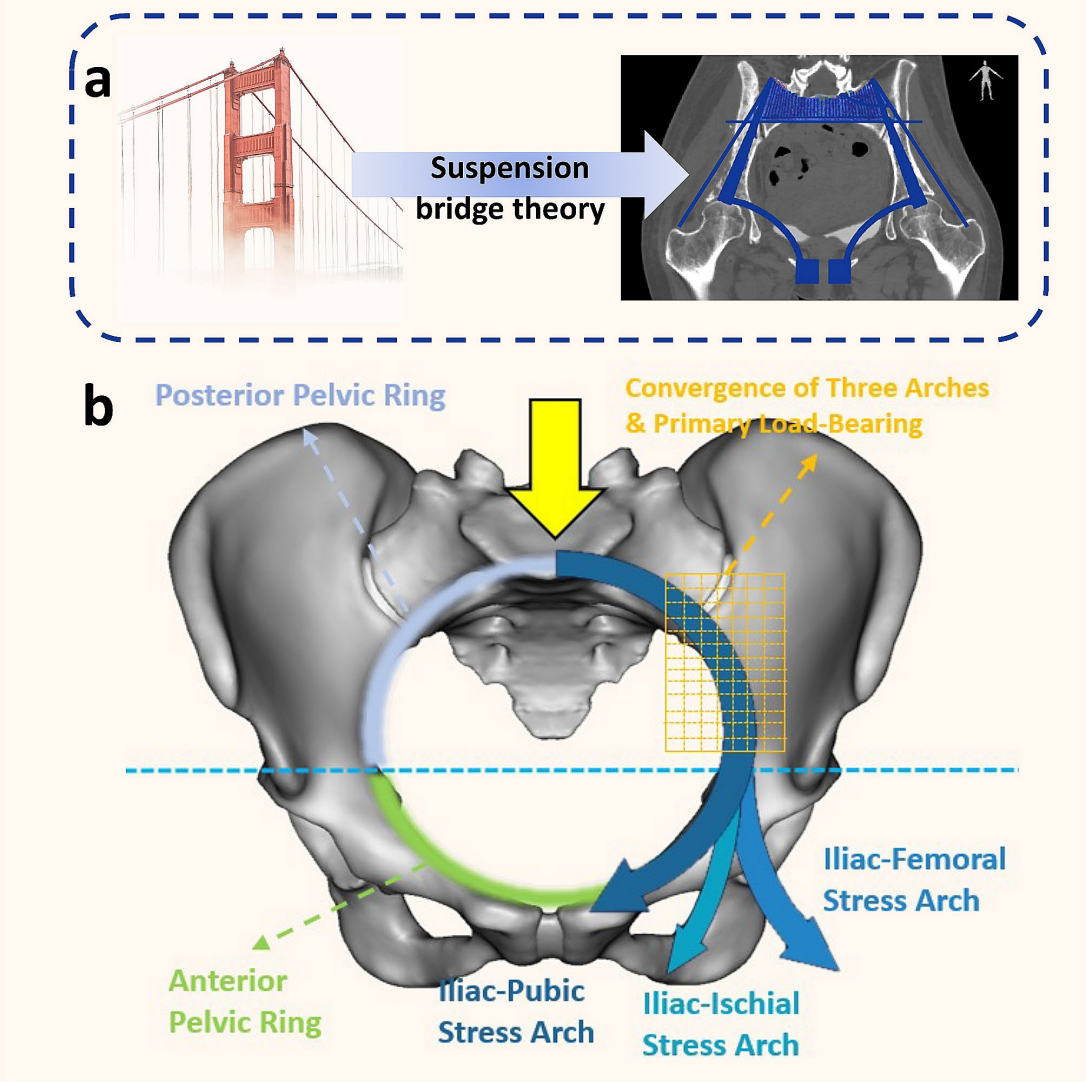
Biomechanical characteristics of pelvic ring stress transmission: **(a)** In the suspension bridge concept, the posterior superior iliac spines are pillars, the interosseous sacroiliac ligaments function as suspension bars, and the sacrum acts as the central bridge. The anterior pelvic ring serves as a pull bar (strut) to prevent lateral spreading and enhance stability. **(b)** The posterior pelvic ring primarily bears weight and serves as the main load-bearing structure, especially at the convergence of three stress arches - iliac-femoral, iliac-pubic, and iliac-ischial stresses - represented by the yellow grid area. This convergence forms the core of weight-bearing. The anterior pelvic ring assumes a secondary load-bearing role, assisting in balancing stress distribution in the posterior ring and contributing to overall stability. The integration and harmony between the anterior and posterior pelvic rings are essential for long-term stability following prosthetic reconstruction. Reprinted with permission from Hu et al. ©2024 International Orthopaedics

Our research proposes a novel classification system for 3D-printed hemipelvic endoprosthetic reconstruction after tumor resection, resulting in favorable functional outcomes (average MSTS score of 76.7%), surpassing comparable investigations [8, 9, 39, 40]. This success is attributed to the following four aspects: (1) Accurate osteotomy facilitated by innovative guide plate designs, tailored to specific surgical approaches and pelvic anatomical landmarks for precise prosthetic implantation. (2) Secure fixation of the prosthetic-bone interface through multiple screws ensures effective initial stability, which is critical for promoting osseointegration and preventing early aseptic loosening of the prosthesis [41]. (3) Effective mid-term stability of the prostheses was achieved through successful osseointegration. All implants featured a porous structure resembling trabecular bone, which is considered to have a promising potential for successful osseointegration [12]. The T-SMART assessment in our study achieved a high success rate of 98.5% (128 out of 130 cases) for bone integration. (4) Theoretically, based on biomechanical research and early clinical follow-up evidence, complete reconstruction of the pelvic ring may contribute to a more balanced distribution of mechanical transmission and potentially enhance long-term prosthetic effectiveness [19, 24, 25, 30, 31, 33, 42–46]. From a biomechanical standpoint, the pelvic ring’s anatomy encompasses static (bony structures) and dynamic (ligaments, muscles, and tendons) stability. The posterior part of this ring structure notably plays a key role in load transfer [47]. However, the anterior pelvic ring also contributes significantly to the overall stability of the system. In the suspension bridge concept, the anterior pelvic ring serves as a pull bar (strut) to prevent lateral spreading and enhance stability (Fig. 5a) [48]. Furthermore, as per Tile [49], the anterior pelvic ring structure and the posterior ring structure contribute 40% and 60% of the overall stability of the pelvic ring, respectively. Therefore, the comprehensive reconstruction of the pelvic ring is beneficial for long-term stability of the prosthetic-host bone system, leading to improved postoperative functionality in patients (Fig. 5b).

The major limitation of our study is the heterogeneity of pathohistological types, but it allows for a larger patient population with pelvis malignancies for a precise evaluation of the classification system. Furthermore, our study is limited by its single-center, retrospective nature and small sample size, impacting data collection and analysis. Larger multicenter studies are needed to evaluate the feasibility of using this classification system in the future. It is essential to acknowledge the short to medium-term follow-up period as a limitation in the study. Therefore, longer follow-up clinical studies are necessary in future research to validate the clinical significance and long-term efficacy of 3D-printed customized hemipelvic prostheses in pelvic girdle reconstruction. In addition, assessing tumor size by measuring the maximum longitudinal, transverse, and vertical diameters of the tumor in MRI and CT images may not be the optimal method for tumor volume assessment. Future research may need to employ three-dimensional image reconstruction or CT or MRI volumetric analysis methods to achieve more accurate measurements.

## Conclusions

3D printing of customized pelvic implants with porous structure offers advantages like improved osteointegration, long-term stability, and precise fit. We formulated a novel classification system based on pelvic defect morphology and 3D-printed hemipelvis endoprostheses. This classification system comprehensively integrates the surgical approach, osteotomy guide plate and prosthesis design, postoperative rehabilitation plans, and the entire perioperative process. It may serve as a valuable supplement to the Enneking and Dunham classification and act as an effective tool for communication among surgeons from diverse disciplines.

### Electronic supplementary material

Below is the link to the electronic supplementary material.

Supplementary Material 1: Figure 1 Typical postoperative complications in hip reconstruction surgery, namely, hip dislocation **(a)** and screw fracture **(b)**. The preoperative pelvic X-ray is shown in **a1** and **b1**, and the X-ray taken three days after Type Ab reconstruction reveals the hip dislocation **(a2)**. Fortunately, successful closed reduction under general anesthesia was performed **(a3)**. **b2** displays the X-ray taken two days after Type Ba reconstruction, and one year postoperatively, a screw fracture **(b3)** at the uppermost part of the sacroiliac joint is evident (marked in red). Notably, the patient remained asymptomatic, and conservative observation was chosen as the management approach. Reprinted with permission from Hu et al. ©2024 Journal of Orthopaedic Surgery and Research.

## Data Availability

The datasets used and/or analysed during the current study available from the corresponding author on reasonable request.

## Abbreviations

3D: Three dimensional
VAS scores: Visual Analog Scale scores
MSTS-93 scale: Musculoskeletal Tumor Society 93 scale
T-SMART: Tomosynthesis Shimadzu Metal Artefact Reduction Technology
