# Supplementary figures and images for: 3D-Printed custom-made hemipelvic endoprosthetic reconstruction following periacetabular tumor resection: utilizing a novel classification system
Source: BMC Musculoskelet Disord. 2024 May 16;25:384. doi: 10.1186/s12891-024-07509-8 (PMC11097426; doi:10.1186/s12891-024-07509-8)

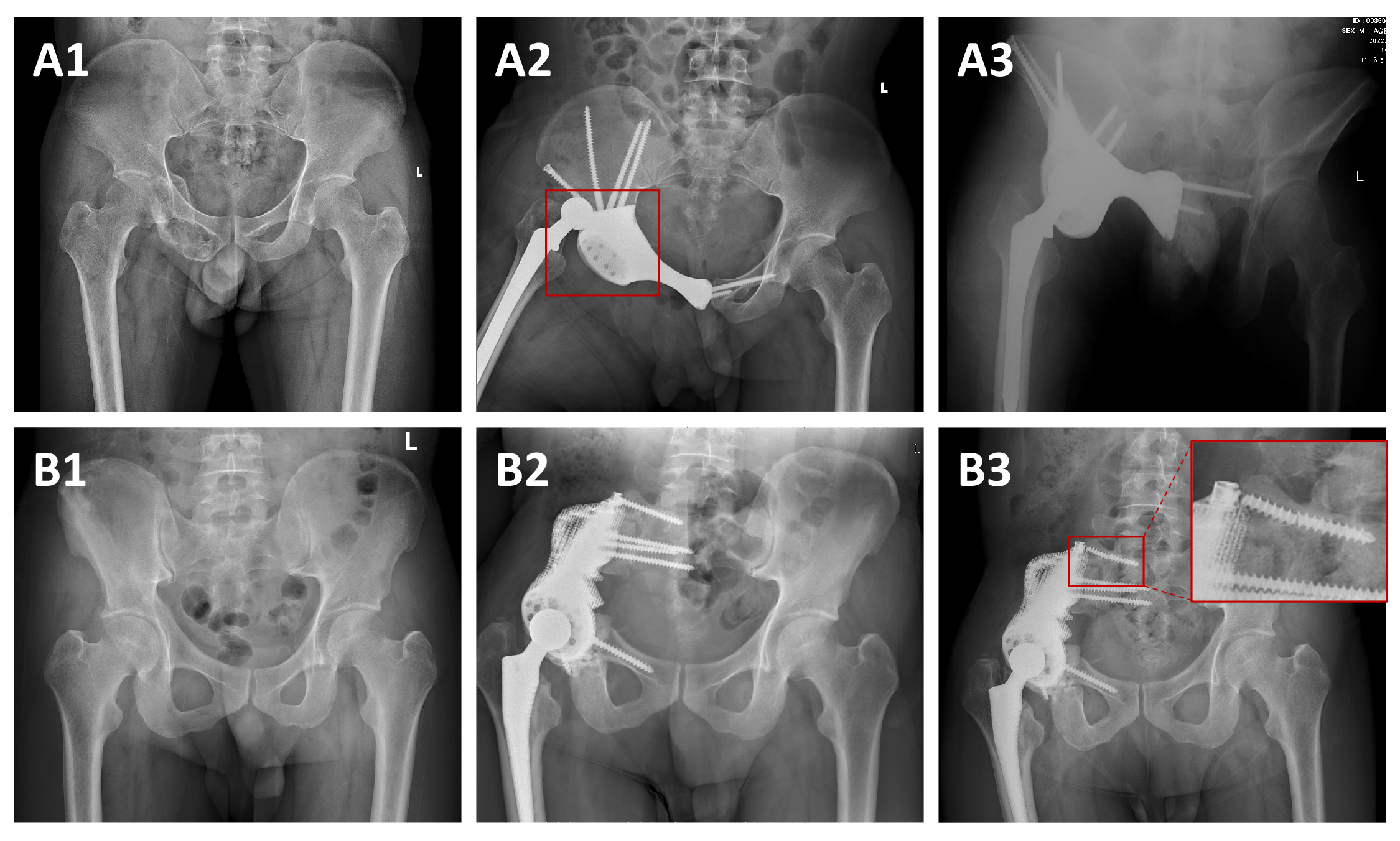

Supplement: Supplementary file 1 — Supplementary Material 1: Figure 1 Typical postoperative complications in hip reconstruction surgery, namely, hip dislocation (a) and screw fracture (b). The preoperative pelvic X-ray is shown in a1 and b1, and the X-ray taken three days after Type Ab reconstruction reveals the hip dislocation (a2). Fortunately, successful closed reduction under general anesthesia was performed (a3). b2 displays the X-ray taken two days after Type Ba reconstruction, and one year postoperatively, a screw fracture (b3) at the uppermost part of the sacroiliac joint is evident (marked in red). Notably, the patient remained asymptomatic, and conservative observation was chosen as the management approach. Reprinted with permission from Hu et al. ©2024 Journal of Orthopaedic Surgery and Research. [file 12891_2024_7509_MOESM1_ESM.tif]
